# Next-generation sequencing of the tonsillar microbiome in severe acute tonsillitis: comparison with healthy controls and culture-based findings

**DOI:** 10.1007/s10096-025-05195-5

**Published:** 2025-06-25

**Authors:** Camilla Andersen, Tine Sneibjerg Ebsen, Casper Aabrandt Thorup, Kasper Basse Reinholdt, Ann Marlene Gram Kjaerulff, Nichlas Udholm, Vesal Khalid, Adnan Madzak, Christophe Duez, Henrik Münch, Søren Pauli, Christian Sander Danstrup, Niels Krintel Petersen, Thomas Greve, Tejs Ehlers Klug

**Affiliations:** 1 Department of Clinical Microbiology, Aarhus University Hospital, Palle Juul-Jensens Boulevard 99, Aarhus, DK-8200 Denmark; 2 Department of Otorhinolaryngology, Head & Neck Surgery, Aarhus University Hospital, Aarhus, Denmark; 3 Department of Otorhinolaryngology, Head & Neck Surgery, Aalborg University Hospital, Aalborg, Denmark; 4 Department of Clinical Medicine, Aalborg University, Aalborg, Denmark; 5 Aarhus University, Aarhus, Denmark

**Keywords:** 16S tNGS, Nanopore, Next-generation sequencing, Pathogens, Acute tonsillitis

## Abstract

**Purpose:**

Previous culture-based studies suggest three significant pathogens in acute tonsillitis (AT): *Streptococcus pyogenes*, *Fusobacterium necrophorum*, and *Streptococcus dysgalactiae*. Next-generation sequencing (NGS) provides further insights into the human microbiome and may pinpoint additional pathogens in bacterial infections. We aimed to investigate the tonsillar microbiome and identify pathogens associated with AT by applying NGS to tonsillar swabs from patients with severe AT, comparing the findings with both healthy controls and culture-based results.

**Methods:**

Full-length sequencing of the 16S rRNA gene (16S tNGS) was performed on tonsillar swabs from 64 AT patients and 55 controls, who were prospectively enrolled at two Danish Ear-Nose-Throat Departments between June 2016 and December 2019.

**Results:**

The mean number of detected bacteria was significantly higher in patients analysed with 16S tNGS (36) than with culture methods (6.5, *p* < 0.001). The alpha diversity was lower in patients compared to controls (*p* < 0.001) and beta diversity showed separation of the two groups (*p* = 0.001). *S. pyogenes* (*p* = 0.001) and *Bifidobacteriaceae* (*p* = 0.002) were significantly more abundant in patients compared to controls. The three suggested pathogens were detected more frequently using 16S tNGS compared to culture: *S. pyogenes* (38% vs. 27%, *p* = 0.26), *F. necrophorum* (19% vs. 11%, *p* = 0.32), and *S. dysgalactiae* (14% vs. 11%, *p* = 0.79).

**Conclusion:**

The tonsillar microbiome differed significantly between AT patients and healthy controls. Our findings confirm the role of *S. pyogenes* in AT, but did not identify additional likely pathogens. The addition of 16S tNGS to cultures increased the collective detection rate of three previously suggested pathogens from 48 to 70%.

## Introduction

Acute tonsillitis (AT) is a very frequent reason for consultation in general practice [1]. The vast majority of patients have uncomplicated, self-limiting disease and they do not require microbiological investigations or antibiotic treatment [2]. A limited number of bacteria, including *Streptococcus pyogenes*, *Fusobacterium necrophorum*, and *Streptococcus dysgalactiae* are considered pathogenic in AT based on studies using culture methodology [3–5, 6, 7]. However, cultures provide incomplete insights to all the potential bacterial pathogens, as some fastidious or fragile bacteria are unable to grow under the provided conditions [8–9]. Recently, significant advancements in genomic technologies, particularly next-generation sequencing (NGS), have enhanced the ability to characterize and analyse the human microbiome [8, 10].

Few studies have investigated the bacterial composition of the microbial community in the head and neck region (including the larynx, the tonsils, the middle ear, and the paranasal sinuses) using sequencing-based methods [8, 10–13]. However, to our knowledge, no microbiome studies have been conducted using tonsillar specimens from patients with AT.

In a previous publication, we reported the bacterial findings in patients with severe acute tonsillitis (AT), who were referred to Ear-Nose-Throat specialists for specialized management, utilizing meticulous and comprehensive culture-based methods [9]. The present study aimed to further investigate the tonsillar microbiome and identify significant pathogens associated with AT by applying NGS to tonsillar swabs from a selected subgroup of patients exhibiting severe symptoms and distinct clinical and biochemical signs of infection, comparing the findings with both healthy controls and culture-based results. We hypothesized that the addition of NGS would detect significant bacterial pathogens in a greater proportion of patients compared to traditional culture-based methods.

## Materials and methods

### Participants and samples

Patients were prospectively enrolled in the period June 2016– December 2019 at two Danish Departments of Otorhinolaryngology, Head and Neck Surgery (Aarhus University Hospital and Aalborg University Hospital). Healthy controls (medical students) were enrolled in the period March– May 2022. Detailed enrolment and sample collection are described in a previous publication [9]. Tonsillar swabs were collected in E-swab media (Copan, Brescia, Italy), and placed at -80 °C within 30 min of collection. Samples were cultured for microbiological analyses, and surplus material was subsequently stored at -80 °C for sequencing analyses.

## DNA extraction

Tonsillar swabs were thawed at room temperature and 500 µL of liquid medium were treated with bead-beating with Precellys Lysing Kit (Bertin Instruments, Montigny-le-Bretonneux, France) using MagNA Pure Tissue Lysis Buffer (Roche Life Science, Basel, Switzerland) plus 50 µL Proteinase K (QIAGEN, Hilden, Germany), and incubated for 1 h at 56 °C. DNA extraction was performed using the DNeasy Blood and Tissue Kit (QIAGEN) following the instructions for Tissue, except using 400 µL of Buffer AL and 400 µL ethanol, washing twice with Buffer AW2, and eluting in 80 µL nuclease-free water. Negative template controls using MagNA Pure Tissue Lysis Buffer and E-swab media were prepared identically to the samples. The DNA was stored at -20 °C.

## PCR

For amplification of the V1-V9 hypervariable region of the 16S rRNA gene, the following primers were used: forward primer: 5’-AGRGTTYGATYMTGGCTCAG-3’ (S-D-Bact-0008-c-S-20) and reverse primer: 5’-CGGYTACCTTGTTACGACTT-3’ (1492R) [14]. PCR amplification of 16S rRNA genes was conducted using the KAPA HiFi HotStart ReadyMix PCR kit (Kapa Biosystems, Wilmington, MA, USA) in a total volume of 25 µL according to the manufacturer’s instructions. Amplification was performed with 30 cycles of 98 °C for 20 s, 68 °C for 45 s and 72 °C for 30 s. Amplified DNA was purified using AMPure^®^ XP magnetic beads (Beckman Coulter, Brea, CA, USA), and quantified using Qubit™ 1X dsDNA BR (Broad Range) Assay Kit and Varioskan™ Lux microplate reader (Thermo Fisher Scientific, Waltham, MA, USA).

## 16S rRNA targeted sequencing on the minion™ platform (16S tNGS)

Library was prepared with the Ligation sequencing amplicons-native barcoding protocol, SQK-LSK109 with EXP-NBD196 (Oxford Nanopore Technologies (ONT), Oxford, UK) according to the standard procedures described by ONT, and with a total input of 200 ng per sample. Finished library was loaded onto the R9 flow cell (FLO-MIN106D, ONT) and full length 16S rRNA amplicon sequencing was done using a MinION™ nanopore sequencer (ONT) for 48 h. MinKNOW software (version 22.05.5) was used to control and monitor sequencing. Live basecalling was done using Guppy (version 6.1.5) with the following settings: High accuracy basecalling, demultiplexing and barcode trimmings. A minimum Qscore of 9 and minimum read length of 200 was used to remove low quality reads.

## Long-read 16S bioinformatics analysis

Quality of reads was assessed using NanoStat (version 1.1.2, https://github.com/wdecoster/nanostat) [15]. Reads were trimmed of adaptors using Porechop (version 0.2.4, https://github.com/rrwick/Porechop). Human reads were removed with minimap2 (version 2.24) [16] and human reference hg38. Taxonomic profiles for each sample were generated with Emu (version 3.4.4) [17], utilising the default parameters and suggested database.

The output tables from Emu were converted into Phyloseq objects (version 1.42.0) [18] and downstream analysis carried out on the phyloseq objects in R (version 4.2.3). Each sample was filtered to exclude species with less than 50 associated reads or a minimum abundance fraction below 0.001. Given that microbial load varies between individuals and changes in the absolute abundance of a single taxon can alter the relative abundance of all taxa, absolute abundances could not be determined [19]. Instead, percentage distribution of reads (relative abundance) was used to visualize community compositions. Alpha and beta diversity analyses were conducted on raw data using tools from the phyloseq package. Specifically, samples were then rarefied to an even depth, and alpha diversity was assessed using the Shannon index, while beta diversity was calculated using the Bray-Curtis dissimilarity distance.

### Cultures

Tonsillar swabs were thawed at room temperature and processed in a class 2 laminar air flow safety cabinet using an aseptic technique. Swabs were vortexed for 5 s and 10 µL of liquid medium was plated on 5% horse blood agar, chocolate agar, Mueller Hinton agar with 5% horse blood and 20 mg/L NAD plus selected antimicrobial discs used for initial differentiation of bacterial species, anaerobic agar (chocolate plate containing K-vitamin and cysteine), and selective *Fusobacterium* agar (containing 5 mg/L nalidixic acid and 2.5 mg/L vancomycin) (Statens Serum Institute Diagnostica, Hillerød, Denmark). Plates were incubated for four days at 35 °C, the first three plates in 5% CO_2_, and the latter two plates in anaerobic atmosphere including a metronidazole-disc (10 UG) (Oxiod, Roskilde, Denmark).

## Comparison of the bacterial composition in AT and controls using culture-based methods and 16S tNGS

A custom taxonomy was developed to compare culture-based findings and 16S tNGS results. This approach was used to align with clinical understanding, even though it may differ from bioinformatics classifications and result in some loss of specificity. Bacteria were grouped at different taxonomic levels, with some kept at species level, others at genus level, and some at family level. Unassigned taxa, along with bacteria associated with the environment or non-human hosts, were grouped under ‘others’. Alpha and beta diversity metrics were employed to visualize and evaluate differences in bacterial composition between patients and controls. The distribution of bacterial reads (using custom taxa) was visualized to highlight differences between groups. To compare methods, the number of samples with positive identification of specific bacteria by culture was contrasted with the number of samples identifying the same taxa through 16S tNGS. These findings were illustrated using pairwise comparisons to demonstrate the agreement and discrepancies between the two methods.

## Identifying key bacterial pathogens associated with AT

Bacteria were considered potentially pathogenic if differential abundance analysis revealed a higher prevalence in AT patients compared to controls. Furthermore, a higher percentage of reads in samples that also tested positive by culture was considered supporting evidence for the significance of the identified pathogen.

### Statistical analysis

Differences in alpha diversity were assessed using the standard Welch Two-Sample t-test. Beta diversity differences were analysed using the *adonis2* function in the *vegan* package for R (version 2.6-4) [20]. Principal Coordinates Analysis (PCoA) was performed to visualize Bray-Curtis dissimilarity matrices.

Analysis of Compositions of Microbiomes with Bias Correction (ANCOM-BC2) was used to compare differential abundance between patients and controls [19]. ANCOM-BC2 was run using default settings, adjusting for smoking. The Holm-Bonferroni method was used to correct for multiple testing in the ANCOM-BC2 model. Statistical analyses were performed using the Fisher’s exact test for categorical variables (sex and smoking), the Student’s *t*-test for continuous variables (age and number of species), and the Kruskal-Wallis test for non-parametric variables (percentage of reads). Statistical significance was defined as *p* < 0.05.

### Permissions

The study is registered in the ClinicalTrials.gov protocol database (Unique Protocol ID: 52683). The study was approved by the Ethical Committee at Aarhus County (# 1-10-72-71-16) and by the Danish Data Protection Agency (# 1-16-02-65-16). Informed consent was obtained from all patients in accordance with the guidelines set by the Danish National Board of Health.

## Results

### Patients and controls

A total of 64 patients with AT and 55 healthy controls were enrolled, with the clinical characteristics detailed in Table 1. Significantly more patients reported smoking (41%) compared to controls (4%) (*p* < 0.001). Antibiotics were prescribed to 32 (50%) of patients prior to admission; the vast majority (29/32) were treated with phenoxylmethyl-penicillin.

**Table 1:** Clinical and biochemical characteristics of 64 patients with acute tonsillitis and 55 healthy controls

	Patients *n* = 64	Controls *n* = 55	*p*
Males	24 (38%)	28 (51%)	0.19
Age, mean (SD)	25.7 (5.6)	26.3 (1.4)	0.44
Antibiotic treatment prior to admission	32 (50%)^1^		
Tobacco smoking (current)	26 (41%)	2 (4%)	< 0.001
Temperature, mean ^o^C (SD)	38.4 (0.8)		
Biochemistry, mean (SD)			
C-reactive protein, mg/L	167.0 (112.7)		
Leukocyte count, x10^9^/L	14.6 (5.0)		
Neutrophil count, x10^9^/L	11.6 (4.7)		
Lymphocyte count, x10^9^/L	1.6 (0.7)		
Number of species, mean (SD)			
Sequencing	36.4 (6.0)	47.1 (4.7)	< 0.001
Culture	6.5 (1.5)	8.3 (1.3)	< 0.001
Match between culture and sequencing	5.0 (1.5)	7.4 (1.4)	< 0.001

^1^ Phenoxymethyl-penicillin (*n* = 29) or amoxicillin-clavulanic acid (*n* = 3)

### OTU (Operational taxonomic Unit) assignment

Sequencing of PCR amplicons generated from the V1-V9 hypervariable regions of the 16S rRNA gene from 119 tonsillar swabs produced 28,163,319 raw sequencing reads. After quality filtering, removal of chimeric reads, and applying a cut-off (minimum 50 reads or abundance fraction > 0.001) 23,434,054 reads were retained, averaging 196,920 reads per sample (range: 99,906 − 414,396). A total of 684 OTUs were assigned using the Emu database and these OTUs were assigned into 13 phyla, 25 classes, 42 orders, 82 families, 223 genera, and 83 custom taxonomies.

### Distribution of the microbial community in patients and controls

**Fig. 1:**
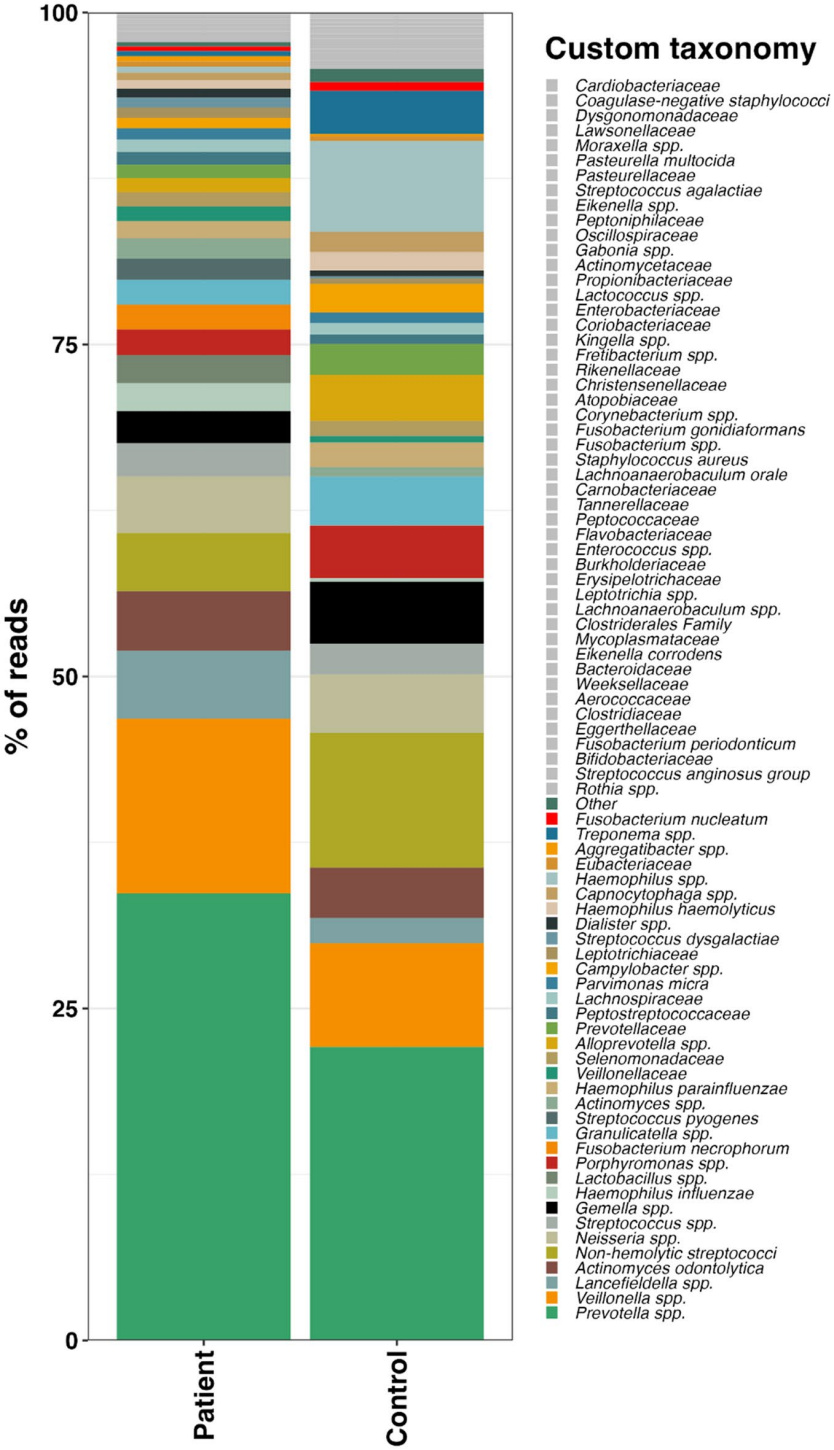
Percentage of reads of bacteria in the tonsils at custom taxonomy level in 64 patients with acute tonsillitis and 55 healthy controls

For both patients and controls, the majority of bacterial taxa fell into two dominant phyla; *Bacteroidetes* and *Firmicutes*. A total of 75 different custom taxa were assigned to patients, while 82 different custom taxa were assigned to controls. The custom taxa Coagulase-negative staphylococci, *Dysgonomonadaceae*, *Cardiobacteriaceae*, *Lawsonellaceae*, *Streptococcus agalactiae*, *Pasteurella multocida*, *Pasteurellaceae*, and *Moraxella* spp. were detected only in controls, whereas *Lactococcus* spp. was detected only in patients (Fig. 1).

### Diversity analysis of microbial communities in patients and controls

**Fig. 2:**
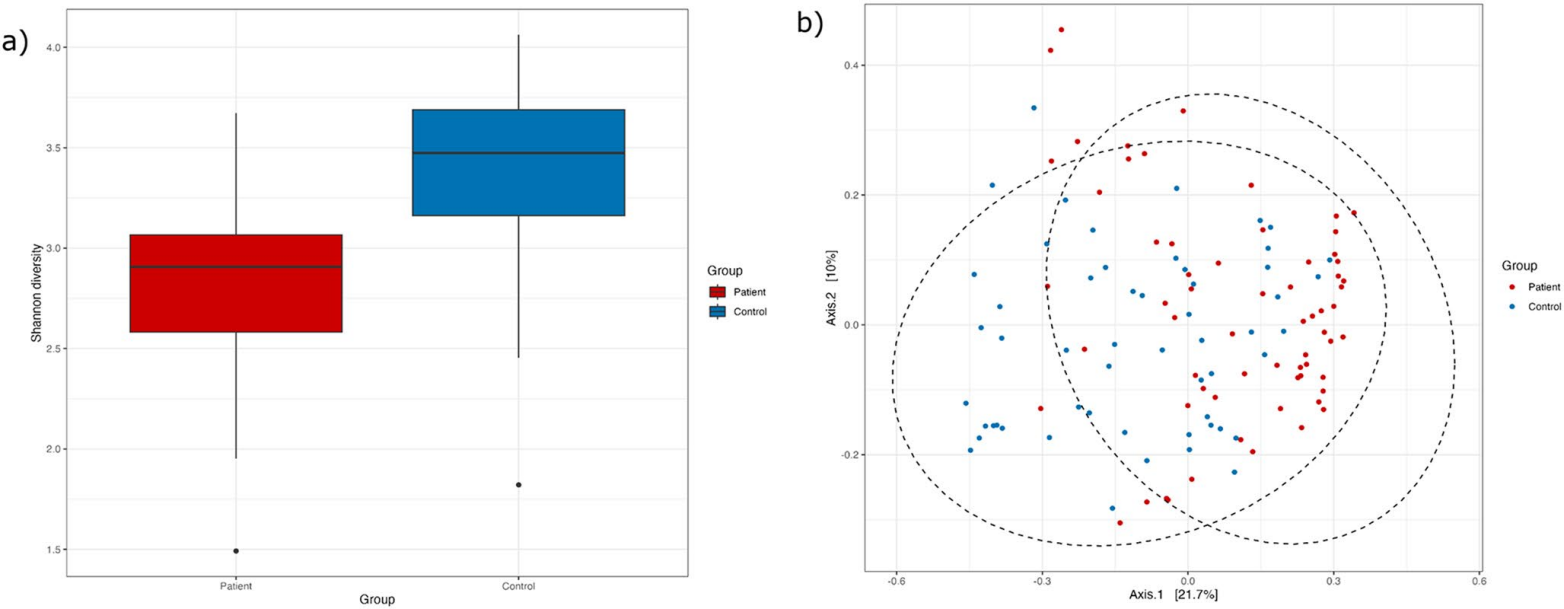
(**a**) Alpha diversity (Shannon index) and (**b**) beta diversity (Principal Coordinate Analysis (PCoA)) comparing 64 patients with acute tonsillitis and 55 healthy controls

The mean number of bacterial taxa detected by sequencing was significantly lower in patients (36.4, SD 6.0) compared to controls (47.1, SD 4.7) (*p* < 0.001). The alpha diversity was significantly lower in patients compared to controls (*p* < 0.001, Welch’s t-test) using the Shannon index (Fig. 2a). Beta diversity showed separation of the two groups with significant differences in the overall structures of the microbial communities between patients and controls (*p* = 0.001, adonis2) (Fig. 2b).

### Microbial taxonomies in patients and controls

**Fig. 3:**
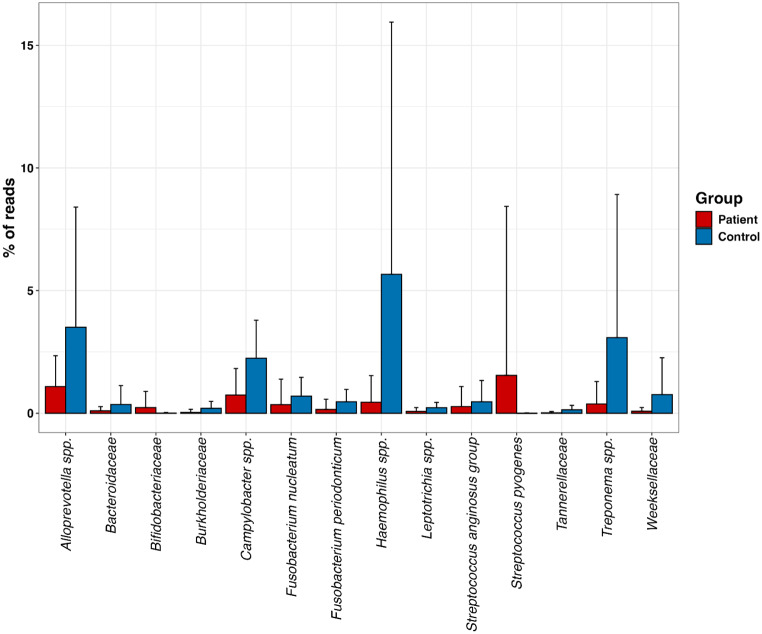
Differential abundance analysis between 64 patients with acute tonsillitis and 55 healthy controls using Analysis of Compositions of Microbiomes with Bias Correction (ANCOM-BC2)

ANCOM-BC2 analysis revealed that two taxa, *Streptococcus pyogenes* (*p* = 0.001) and *Bifidobacteriaceae* (*p* = 0.002), were significantly more abundant in patients compared to controls. In contrast, 12 taxa were more abundant in controls than in patients, including *Alloprevotella* spp. (*p* = 0.035), *Bacteriodaceae* (*p* = 0.021), *Burkholderiaceae* (*p* = 0.001), *Campylobacter* spp. (*p* = 0.022), *Fusobacterium nucleatum* (*p* = 0.001), *Fusobacterium peridonticum* (*p* < 0.001), *Haemophilus* spp. (*p* < 0.001), *Leptotrichia* spp. (*p* = 0.002), *Streptococcous anginosus* group (*p* = 0.032), *Tannerellaceae* (*p* < 0.001), *Treponema* spp. (*p* < 0.001) and *Weeksellaceae* (*p* < 0.001) (Fig. 3).

Two additional taxa were revealed in a subgroup analysis excluding patients with cultures positive for known pathogens (*S. pyogenes*, *F. necrophorum*, and *S. dysgalactiae*). *Lactobacillus* spp. was significantly more abundant in patients compared to controls (*p* = 0.034), whereas *Gemella* spp. was more abundant in controls (*p* = 0.044).

### Comparison of sequencing and cultures in patients

**Fig. 4:**
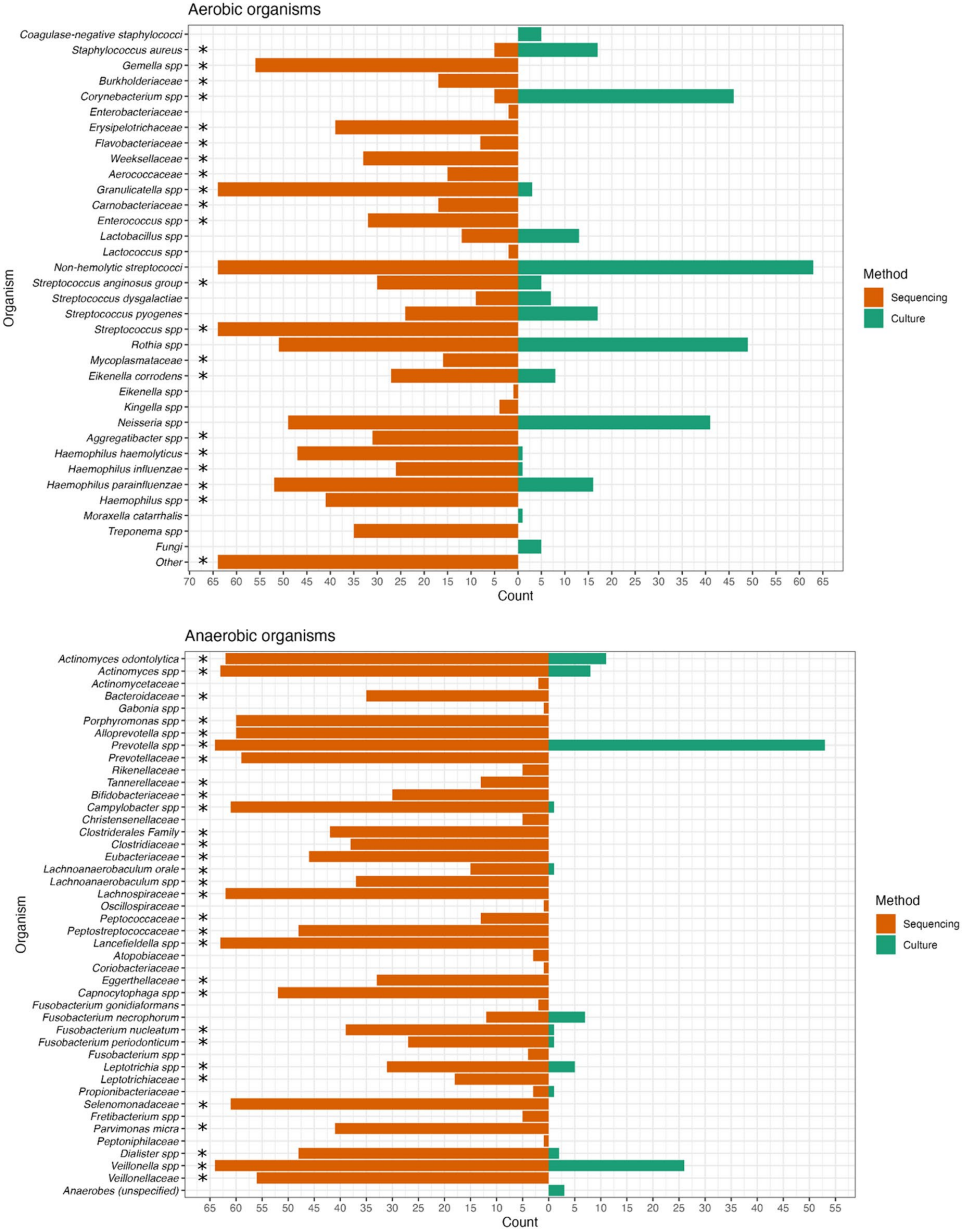
Methodological comparison of 16S tNGS and culture of tonsillar surface swabs in 64 patients with acute tonsillitis. Statistical comparison has been made for bacteria considered culturable on the included media used for culture (*p < 0.05, Fisher’s exact test)

Sequencing analysis detected a total of 75 different custom bacterial taxa, compared to 30 different bacteria using culture-based methods (Fig. 4). Fungi were cultured from five patients; however, no fungal sequences were retrieved, as the sequencing method targeted only prokaryotic 16S rRNA amplicons.

Thirty anaerobic bacteria were identified in significantly higher proportions by sequencing than culture, including the culturable anaerobes: *Prevotella* spp. (sequencing: 64/64 vs. culture: 53/64, *p* < 0.001, Fisher´s exact test), *Veillonella* spp. (64/64 vs. 26/64, *p* < 0.001), *Actinomyces* spp. (63/64 vs. 19/64, *p* < 0.001) and *Leptotrichia* spp. (31/64 vs. 5/64, *p* < 0.001) (Fig. 4).

Nineteen aerobic bacteria were identified in significantly higher proportions by sequencing than culture, whereas the two aerobes *Corynebacterium* spp. (5/64 vs. 46/64, *p* < 0.001) and *Staphylococcus aureus* (5/64 vs. 17/64, *p* = 0.009) were identified significantly less frequently using sequencing (Fig. 4).

Two aerobic bacteria, associated with the commensal pharyngeal flora, were detected at similar frequencies by both sequencing and culture, thus being statistically non-significant: non-hemolytic streptococci (64/64 vs. 63/64, *p* = 1.00) and *Rothia* spp. (51/64 vs. 49/64, *p* = 0.83).

The three bacteria previously identified as pathogens in AT were more frequently detected by sequencing than culture, though not statistically significant: *S. pyogenes* (24/64 vs. 17/64, *p* = 0.26), *F. necrophorum* (12/64 vs. 7/64, *p* = 0.32), and *S. dysgalactiae* (9/64 vs. 7/64, *p* = 0.79). Comparing the combined identification of the three pathogens, significantly more patients were identified with a pathogen using sequencing (45/64, 70%) than culture (31/64, 48%) (*p* = 0.019).

### Percentage of reads for *F. necrophorum*, ***S. pyogenes*** and *S. dysgalactiae* in patients

The median percentages of reads for *S. pyogenes*, *F. necrophorum*, and *S. dysgalactiae* in tonsillar swabs from AT patients are summarized in Table 2.

**Table 2:** Percentages of reads of three tonsillar pathogens in tonsillar surface swabs from 64 patients with acute tonsillitis. Numbers are medians in % (range) in sequencing analysis

	Detected in sequencing and cultures	Detected in sequencing only	*p*
*Fusobacterium necrophorum*	7.64 (< 0.1-16.24)	0.26 (< 0.1-14.66)	0.68
*Streptococcus pyogenes*	0.49 (< 0.1-49.72)	< 0.1 (< 0.1–0.1)	0.004
*Streptococcus dysgalactiae*	1.63 (< 0.1-31.57)	< 0.1 (-)	0.074

< 0.1: present in sample (reads > 50), but with an abundance lower than cut-off (abundance < 0.001)

The percentage of reads was generally low in sequencing-positive samples that were culture-negative. In contrast, double-positive samples showed higher percentages of reads compared to sequencing-only samples, with statistically significant differences observed for *S. pyogenes* (*p* = 0.004), but not for *F. necrophorum*, (*p* = 0.074) and *S. dysgalactiae* (*p* = 0.68).

### Concordance between sequencing and cultures at sample level

At sample level, the number of bacteria detected by sequencing (mean 36.4, SD 6.0) was significantly higher than that identified by culture (6.5, SD 1.5) (*p* < 0.001). The mean number of bacteria detected by both methods was 5.0 (SD 1.5) (Table 1).

## Discussion

We studied a highly selected group of 64 patients with severe AT, who were referred to Ear-Nose-Throat specialists for specialized management. These patients exhibited substantial symptoms of AT, clear clinical signs of infection, and markedly elevated C-reactive protein and neutrophil levels, suggestive of bacterial infection. In a previous study, we reported the microbiological findings from cultures in this same patient group [9]. Our results suggested pathogenic significance for *S. pyogenes*, *F. necrophorum* and *S. dysgalactiae*. However, we were surprised to note that 52% of patients were culture-negative to these pathogens and, hence, without microbiological evidence for bacterial infection despite the use of meticulous and comprehensive culture methods. This observation led us to initiate the present study, assuming that more comprehensive characterization with a sequencing-based method would provide more insight to the potential pathogens in AT.

### Suggested significant pathogens

We found that the tonsillar microbiome was markedly different in patients with AT compared to healthy controls with decreased alpha diversity and separation in beta diversity as well as a lower number of individual bacteria in infected tonsils. *S. pyogenes* was found in higher proportion in AT compared to controls, confirming the well-described pathogenicity of this bacterium. *Bifidobacteriaceae* was the only other bacterium with significantly higher abundance in patients than controls. To our knowledge, *Bifidobacteriaceae* has not previously been linked to pharyngeal infections and it is very rarely implicated in human infections. Further studies are needed to clarify its potential role in AT. Given the large number of analyses conducted, this finding may be incidental. Alternatively, the higher abundance of *Bifidobacteriaceae* could be a secondary effect of other microbiome diversity alterations. Therefore, we interpret our results as indicating no additional likely pathogens in AT. Similar considerations apply to our finding of a higher abundance of *Lactobacillus* spp. in the subgroup analysis of patients with negative cultures for known pathogens.

While sequencing detected a higher proportion of patients (45/64, 70%) with one of the three previously suggested pathogens (*S. pyogenes*, *F. necrophorum* and *S. dysgalactiae*) compared to cultures (48%), the majority of the additional positive findings were present in very low abundances and they may not be clinically significant. 50% of patients received antibiotics prior to admission, and patients with no suggested pathogens using culture were more frequently treated with antibiotics prior to admission (64% vs. 32%, *p* = 0.014). This may explain the relatively low prevalence of pathogens in cultures. However, we expected DNA amplification to yield a higher number of positive samples in patients with recent antibiotic exposure than was observed in the current study. Some patients may have had viral infections, potentially explaining the absence of detectable pathogenic bacteria. However, markedly elevated infection markers, frequent peritonsillar involvement, severe symptoms, and rapid recovery with antibiotics argue against a purely viral cause, though it cannot be ruled out entirely. Another possibility is a co-infection with a virus and less pathogenic bacteria, which may have gone undetected in our relatively small patient cohort.

We identified twelve bacteria with decreased abundance in AT compared to controls. Whether a lack of certain protective bacteria is primary in the development of infection or is a secondary effect of pathogen overgrowth remains unclear.

Our findings suggest that routine sequencing of tonsillar swabs in AT provides limited additional clinical value compared to culture-based methods.

#### Comparison of sequencing and cultures

A larger number of taxa containing known anaerobic bacteria were detected through sequencing compared to culture, highlighting the challenges associated with culturing anaerobes. In contrast, certain Gram-positive bacteria, including *Corynebacterium* spp., *S. aureus*, and Coagulase-negative staphylococcus, were more frequently detected by culture than sequencing. This discrepancy may be attributed to the choice of DNA extraction method (see limitations).

### Previous studies of the tonsillar Microbiome using sequencing-based methods

Only three previous studies have been conducted exploring the bacterial findings in patients with acute pharyngeal infection using sequencing-based methods [11, 13, 21]. Atkinson et al. investigated throat swabs from 341 adults aged 15–30 years with sore throat symptoms and 30 age-matched healthy controls [11]. They amplified the V4 region of the 16s rRNA gene, restricting the analysis to *F. necrophorum* and *S. pyogenes*. The authors concluded that *F. necrophorum* appeared to play a dominant role in sore throat among adolescents and young adults. Yeoh et al. collected oral rinse samples from 43 patients admitted to hospital with AT and 165 healthy controls [13]. Amplifying the V3-V4 variable regions of the 16S rRNA gene, the authors concluded that the oral cavity microbial community was altered during AT. The differences between groups were subtle and restricted to associations between a number of *Prevotella* taxa to AT and several *Neisseria* sequence variants to non-infection. Consumption of antibiotics did not influence the bacterial composition, but smoking was associated with enrichment of several *Fusobacterium* variants. Saar et al. applied sequencing of the V3-V4 region of the 16S rRNA gene to tonsil biopsies and pus samples from 91 patients with peritonsillar abscess [21]. The most abundant species in both sample types were *S. pyogenes*, *F. necrophorum*, and *Fusobacterium nucleatum* leading the authors to conclude that these were the most likely causative pathogens. In contrast, we found *F. nucleatum* to be more abundant among controls than patients. The absence of healthy controls, and the use of tonsillar biopsies and pus samples, in the study by Saar et al. limits the ability to determine whether the identified bacteria represent true pathogens or constitute remnants of the normal tonsillar flora.

In addition, two studies describe the tonsillar microbiome in different groups without clinical infection at time of specimen collection [10, 12]. Jensen et al. investigated tonsillar swabs from 20 subjects (children with tonsillar hyperplasia (*n* = 5) or recurrent tonsillitis (RT, *n* = 5), healthy adults (*n* = 5), and adults with RT (*n* = 5)), using a combination of an in-depth 16s rRNA gene pyrosequencing approach and phylogenetic analysis with detection of species-specific sequence signatures [12]. RT was associated with a shift in the microbiota of the tonsillar crypts in unifrac analysis. *F. necrophorum*, *Streptococcus intermedius* and *Prevotella melaninogenical/histicola* were associated with RT in adults, whereas species traditionally associated with acute tonsillitis (including *S. pyogenes*) were scarce. Watanabe et al. amplified the V4 region of the 16S rRNA gene to investigate tonsillar swabs from 99 patients; 48 adults with immunoglobulin A nephropathy (IgAN), 21 adults with RT and 30 children with tonsillar hyperplasia [10]. Though the diversity in the bacterial composition was substantial in each sample, authors noted that similar patterns of bacteria were present in patients with IgAN and RT, suggesting that the host response to the bacterial flora might be important in the development of IgAN.

#### Limitations

Samples were freeze-thawed for culture prior to sequencing, which may have altered the microbiota profile. Additionally, half of the patients had received antibiotics before sampling, potentially affecting bacterial composition and distribution of reads.

DNA extraction methods vary in effectiveness, particularly in lysing of microorganism, with some techniques demonstrating reduced recovery of Gram-positive bacteria compared to Gram-negative. While no consensus exists on the best extraction method, we applied a standardized protocol across all samples [22–23]. To address DNA extraction challenges, we implemented a low cut-off of 50 reads or an abundance fraction of > 0.1%, ensuring that culture-positive samples were generally detected through sequencing.

To account for host DNA, which can impact bacterial sampling, we established a minimum of 100,000 reads per sample after host DNA filtering.

The risk of contamination during the analytical process of samples was controlled by adding negative template controls to the extraction step, the PCR amplification step, and the sequencing step.

We amplified and sequenced full length 16S rRNA amplicons, thus providing more optimal information on taxa. Oxford Nanopore Technologies (ONT) was selected over other sequencing platforms due to its longer reads, which improve species-level classification compared to short-read technologies like Illumina [24]. The higher error rate associated with nanopore sequencing is offset by the longer reads. It is important to note that ONT sequencing excludes large public databases such as SILVA (https://www.arb-silva.de/) and NCBI (https://www.ncbi.nlm.nih.gov/), which are optimized for short reads. Taxonomic assignment is dependent on the database used, meaning rare bacteria absent from the reference database may not be reported. However, as we focused on well-established human pathogens, the risk of missing important bacteria seems low.

We disregarded the shotgun metagenomics because of the high proportion of host DNA in surface swab samples. Furthermore, when sequencing tonsil surface swab samples, which in disease state may harbour both pathogenic bacteria and those that are part of the commensal pharyngeal flora, the identification of pathogens can be challenging, as all DNA is amplified.

To correct for sampling bias, we used ANCOM-BC2 for differential abundance analysis, a conservative method that minimizes false discoveries [19, 25].

## Conclusion

The tonsillar microbiome in patients with AT differed significantly from that of healthy controls, showing decreased alpha diversity and distinct separation in beta diversity. Our findings confirm the role of *S. pyogenes* in AT, but did not identify additional likely pathogens through 16S tNGS. While the addition of 16S tNGS to culture-based methods increased the collective detection rate of the three previously suggested pathogens (*S. pyogenes*, *F. necrophorum* and *S. dysgalactiae*) from 48 to 70%, most of the additional detections were in low abundances. Therefore, we do not recommend incorporating sequencing together with a culture-based method into routine processing of tonsillar swabs from patients with AT.

## Data Availability

Anonymized data can be obtained from the corresponding author upon request.
